# Characterizing factors influencing adherence to surgery and chemotherapy amongst women suffering from breast cancer in Mbingo Baptist Hospital Cameroon

**DOI:** 10.11604/pamj.2022.43.102.28672

**Published:** 2022-10-25

**Authors:** Nsom Gwendoline Nkenu, Marius Nsoh, Axler Jean Paul, André Nkondjock

**Affiliations:** 1 Catholic University of Central Africa, Department of Public Health, School of Health Sciences, Yaoundé, Cameroon,; 2 Cameroon Baptist Convention Health Services, Mbingo Baptist Hospital, Oncology Unit, Bamenda, Cameroon,; 3 Georgetown University, Centre for Global Health Practice and Impact, TIDE Project, Yaoundé, Cameroon,; 4 State University of Haiti, Faculty of Medicine and Pharmacy, State University Hospital of Haiti, Port-au-Prince, Haiti,; 5 Ministry of Defense, Military Health Department, Yaoundé, Cameroon,

**Keywords:** Breast cancer, surgery, chemotherapy, adherence, Cameroon

## Abstract

**Introduction:**

Breast cancer (BC) is the most prominent cancer in females and is cited as a leading cause of cancer-related mortality in women worldwide. We aimed to determine factors influencing adherence to surgery and chemotherapy in women with BC in Mbingo Baptist Hospital (MBH).

**Methods:**

we conducted a cross-sectional study in the MBH-Cameroon. Purposeful sampling was used to select participants for investigation. A structured questionnaire was applied to 82 women on chemotherapy in the oncology unit. Socio-demographic, clinical, and therapeutic data were collected from participants. The Morisky Medication Scale (MMS) was used to assess the patient's motivation and knowledge while the Adherence Starts with Knowledge (ASK12) questionnaire was used to measure the patient's barriers to treatment. SPSS was used for data analysis.

**Results:**

the mean age was 46.37 (SD 11 years). Most participants (67.1%) were in the group of (25-50 years). The majority (75.6%) of respondents attended at least primary school. The results showed that adherence to surgery and chemotherapy is low 44% and 56.1% respectively, and this was greatly influenced by treatment delay (P = 0.034), missed chemotherapy dose without medical indication (P=0.029), patient's motivation, and knowledge towards their disease and treatment (P=0.0001 and P=0.0001), respectively.

**Conclusion:**

our results revealed that adherence to surgery and chemotherapy among women with BC in MBH is low and is driven by the patient´s motivation, knowledge about the disease, and treatment.

## Introduction

Breast cancer is a disease in which cells in the breast grow out of control. In 2015, there were an estimated 17.5 million cancer cases and 8.7 million deaths, among different kinds of cancers, breast cancer ranked the most prominent [1]. Globally, approximately 2.1 million diagnoses were estimated in 2018, contributing to about 11.6% of total cancer incidence with approximately 627 000 deaths making 6.6% of total cancer mortality [2]. Though there is a greater incidence of BC in developed countries, low-income countries contribute up to 60% of this death toll [3]. In Cameroon, BC accounts for 18.5% of all cancers in the community and it is the first cancer affecting women with a frequency of 30% [4] the general trends show diagnosis at stage III and IV [5]. Although most patients present with localized BC and can be rendered disease-free with locally endemic therapy, distant metastasis is common and is the primary cause of death from the disease [6]. Adjuvant systemic therapies are effective in reducing the risk of distant and local recurrence, including chemotherapy. Despite a life-threatening diagnosis like BC, patients may not adhere to medication representing a potential missed opportunity for health gain and waste of resources [7].

Treatment adherence is defined by the World Health Organization as “the extent to which a person's behavior, taking medication, following a diet, or executing lifestyle changes, corresponds with the agreed recommendation from a health care provider. Cancer medication non-adherence has been shown to lead to decreased survival, higher recurrence, treatment failure rates, and health care costs [8] Adherence to BC treatment is crucial to obtain optimal health outcomes, such as cure or improvement in the quality of life. Although most BC patients present late at diagnosis, survival rates can be greatly improved with improvement in adherence to recommended treatment. In a Nigerian-based study, patients' adherence and reasons for non-adherence to chemotherapy were evaluated and the results showed that 80.9% of the clients were non-adherent with the most common reason for non-adherence being financial barriers [9]. Another study revealed that 47.9% of BC patients refused a mastectomy and 38.6% adherence to chemotherapy [5]. Increasing the effectiveness of adherence interventions may have a far greater impact on the health of the population than any improvement in specific medical treatments [10]. We aimed to determine the factors influencing adherence to surgery and chemotherapy in women with breast cancer in MBH Cameroon.

## Methods

This study was carried out in Mbingo Baptist Hospital (MBH) oncology unit. The city of Mbingo is located about 25kms from Bamenda the Regional headquarters of the North West Region, Cameroon. It has an estimated population of over 200,000 inhabitants and MBH is a specialized hospital that receives patients from all over the country as well as other countries.

**Study design and data collection:** we conducted a cross-sectional study from January 2018 to December 2018. Data were collected using a structured questionnaire to obtain data on socio-demographic, and clinical characteristics of patients, and adherence to treatment outcomes. We also explored clients' hospital records to further obtain clinical data. The Modified Morisky Scale (MMS) was used to assess the patient's knowledge and motivation towards BC and treatment. It is a six-question scale with yes or no answers. For question 1,2,3,4 and 6, No=1 and Yes=0. For question 5, Yes=1 and No=0 [11]. The scores were summed for the knowledge and motivation column. A score of 0-1 implies low motivation and low knowledge while a score of 2-3 implies high motivation and high knowledge. To score the ASK-12, each subscale score is computed as the sum of all items within that scale, and the total score is the sum of all 12 items. If a participant did not complete 1 item within a subscale, the subscale score was computed by substituting the mean of the available responses for the missing response. If more than 1 item within a subscale was missing, that subscale score should not be computed. Women suffering from cancer who gave their free and voluntary consent were enrolled while women with incomplete hospital records and women on palliative care were excluded.

**Data management and analysis:** data were entered and analyzed using SPSS 20. Descriptive statistics with measures of central tendencies were showed. Chi^2^ test were used to establish the relationship between the dependent variable (adherence to BC treatment) and the independent variables; clinical-stage, BC treatment motivation, and knowledge.

Ethical clearance was obtained from the school of Health Sciences Institutional Review Board N° 2018/0749/CEIRSH/ESS/MSP Catholic University of Central Africa, and the IRB of the Cameroon Baptist Convention Health Board (IRB) NO IRB2018-044. Consent/assent forms were also presented to participants before the start of the study. They were informed explained the objective of the study, benefits, risk, and confidentiality of the information provided. Written consent was obtained before enrollment into the study.

**Patient involvement:** women suffering from breast cancer were not involved in setting the research design and, but they were intimately involved in providing lived experiences. Patients and their families were also central to the dissemination of the baseline information, which helped to generate the information required to realize this study.

## Results

**Socio-demographic and clinical characteristics:** from January 2018-August 2018, a total of 82 women with BC receiving treatment at the MBH oncology unit were recruited and enrolled in the study. The mean age was 46 (SD= 11) years. The age range was (25- 80 years). More than 2/3 of the study participants (67.1%) ranged between (25 and 50 years). About 3/4 (75.6%) of respondents attended at least primary school, 65.9% of participants earned less than 50.000Frs as income per month with only 6.1% of all participants enrolled in a health insurance scheme. The findings showed, (70.7%) of participants were delayed for 1-6 months before starting treatment. The majority (91.5%) of participants reported having BC for their first time and (80.5%) revealed a sporadic unset of cancer. Table 1 describes the participant's socio-demographic and clinical characteristics.

**Table 1:** socio-demographic and clinical characteristics of the study population

Variables	Modality	Frequency	Percentage (%)
Age Group	[25-50]	55	67.1
	[50-80]	27	32.9
Health insurance scheme	Yes	5	6.1
	No	77	93.9
Treatment delay	Less than 6 months	58	70.7
	6 months and above	24	29.3
History of recurrence	Yes	7	8.5
	No	75	91.5
Family history of BC	Yes	16	19.5
	No	66	80.5
Topography of the tumor	Left	41	50
	Right	37	45.1
	Bilateral	4	4.9
Lymph node involvement	Yes	56	68.3
	No	26	31.7

Our findings showed many patients were affected on the left breast (50%) in contrast to the right breast (45.1%). Most of the patients had stage III and stage IV disease (76%) while (24%) had stage I and II disease. Most of the patients had lymph node involvement (68%).

The study showed no significant association between the socio-demographic characteristics and adherence, although a borderline relationship was apparent between educational attainment and adherence (P=0.051). Patients who started treatment early (within the first 3 months) were more likely (P=0.034) to adhere to treatment than patients who started treatment late (above 6 months of diagnosis) (Table 2).

**Table 2:** socio-demographic and clinical characteristics influence on adherence to chemotherapy

Variables	Modality	Likely to adhere (%)	Unlikely to adhere (%)	P-value
Age group	[25;50]	69.60	63.90	0.587
	[50;80]	30.40	36.10	
Religion	Christian	89.10	83.30	0.512
	Muslim	6.50	13.90	
	No religion	4.30	2.80	
Marital status	Leaving in union	63.00	63.90	0.937
	Single	37.00	36.10	
Level of education	No education	23.90	25.00	0.051
	Primary	30.40	50.00	
	Secondary	37.00	11.10	
	Tertiary	8.70	13.90	
Health insurance	Yes	8.70	2.80	0.266
	No	91.30	97.20	
Treatment delay	<months	65.2	36.1	0.034*
	>months	17.4	19.4	
	6 months - 1 year	8.7	27.8	
	>1 year	8.7	16.7	
History of recurrence	Yes	10.9	5.6	0.393
	No	89.1	94.4	
Family history of BC	Yes	15.2	25.0	0.26729
	No	84.8	75.0	
Topography of the tumor	Left	45.7	55.6	0.564
	Right	47.8	41.7	
	Bilateral	6.5	2.8	
Clinical stage	Stage I	8.7	11.1	0.985
	Stage II	15.2	13.9	
	Stage III	47.8	47.2	
	Stage IV	28.3	27.8	

* =statistically significant

Patients who missed a dose of chemotherapy without any medical indication were more unlikely (P=0.029) to adhere to chemotherapy than their corresponding counterparts. Patients with high knowledge and motivation were more likely to adhere to treatment than patients with low knowledge and motivation (P= 0.0001 and P=0.0001) respectively (Table 3).

**Table 3:** therapeutic aspect and adherence to chemotherapy

Variables	Modality	Adhere (%)	Non-adhere (%)	P-value
Missed chemo dose	Yes	15.2	36.1	0.029*
	No	84.8	63.9	
Knowledge	Low Knowledge	17.40	72.20	0.0001*
	High Knowledge	82.60	27.80	
Motivation	Low Motivation	15.20	69.40	0.0001*
	High Motivation	84.80	30.60	

* =statistically significant variable

## Discussion

We assessed adherence to surgery and chemotherapy and its related factors amongst women with BC in MBH. These findings showed a mean age of 46.7 years and range 25-80 years Table 1. These results correlate with findings conducted in Eastern Nigeria showing a mean of 45.2 years (range 18-80 years) [12]. Treatment delay significantly influences adherence patterns (P=0.034), missed chemotherapy dose without medical indication (P=0.029), patient motivation (P=0.0001), and knowledge (P=0.0001) towards their disease and treatment as seen in Table 2. Though no significant relationship existed between age and adherence. A slightly significant result was observed with the patient´s educational level (P=0.051) Table 2. From the participants enrolled in this study, more than 64.6% completed at least primary school Table 2. The level of education influenced understanding the disease and determine how adherence to treatment Table 2. This is correlated with findings that sought to examine the association of patients with adherence to prescribed BC adjuvant chemotherapy and delays in treatment among African American women [13]. This study revealed that educational status significantly influenced total treatment delay (P=0.0001) and therapeutic outcomes Table 3. The associated cost of treatment influenced the ability to diligently stick to treatment. About (93.9%) of participants were not enrolled in a health insurance scheme Ta. Lack of health care coverage can be a barrier to seeking medical care, especially in a poor population and during hard economic times. A significant proportion of the population of Cameroon earns less than a dollar a day and 30% of the population lives below the poverty line [14].

This, coupled with an increasing failure of the extended family network, can make payment for chronic ailments such as cancer, quite challenging. The study did not show any significant relationship between health insurance and adherence. Our study revealed that 43.9% of the patients declined a mastectomy. Using the ASK12 adherence questionnaire, 56.1% of patients were likely to adhere to chemotherapy. This result is different from the results of a similar study carried out by [15] where the proportion of non-adherence to surgery and chemotherapy was 14% and 30.1% respectively. These differences in rates of adherence could be due to differences in the approach of care and public awareness on BC as seen in Table 2. It is similar to the results of a study carried out in Nigeria where 47.9% of patients refused the surgery and 38.6% were adherent to chemotherapy [5].

We found out that 70.7% of the study participants delayed for 1-6 months before starting treatment while 29.3% delayed for 6 months to 1 year before starting treatment. This could be because when BC is diagnosed, patients do not believe in the severity of the disease and only tend to accept recommended treatment when symptoms get worse. From statistical analysis, there was a significant relationship between treatment delay and adherence (P=0.034). Patients who delayed for more than 6 months before starting treatment were more unlikely to adhere to treatment. This could be because beyond 6 months of diagnosis, symptoms get worse and the ability to bear the treatment side effects reduces. This is related to the results of a previous study to determine the impact of treatment delay on prognosis where it was reported that total delays of 3-6 months are associated with lower survival [16]. The study showed that 50% of the patients were affected on their left breast while 41.7% were affected on their right breast and 4.9% had bilateral disease. Similar to Essiben results were (55.7%) of patients had a left breast tumor. We found that most patients (75.6%) were diagnosed at a locally advanced stage (stage III and stage IV) of the disease and axillary lymph nodes were involved in 68.3% of cases. This could be due to poor knowledge of the disease in the population (knowledge of early warning signs and diagnosis such as breast self-examination) as well as poor Public Health awareness and screening programs. These findings are related to the findings of a study carried out in Cameroon which showed that most patients were diagnosed at a locally advanced stage (T4 and T3) of the disease (71.8%) [4].

Another study carried out in Eastern Nigeria had similar results where 72% had advanced stage BC (stages III or IV) [12]. No significant relationship was observed between the clinical stage and adherence to treatment. This is similar to the findings of a study carried out to better understand the specific reasons for acceptance or refusal of chemotherapy in older adults with cancer which showed that the stage of cancer had no significant association with the patient's decision to accept or refuse chemotherapy [17]. While this study did not include data on early detection methods such as breast self-examination, the high percentage of patients who presented with advanced-stage breast cancer suggests the need for culturally sensitive public education on BSE as well as targeted population screening. Our findings revealed that chemotherapy was often first-line therapy (52.4%). This could be due to the advanced stage of the disease at the presentation where surgery (mastectomy) may not be implicated. This is related to other findings where chemotherapy was the first-line therapy in 43.8% of the patients [4]. A low rate of radical mastectomy was observed (56.1%). Of those who gave reasons for refusal of mastectomy, the most common reason was body image (44.4%) followed by fear of surgery 30.6%. We can explain using the HBM that the patients who refused radical mastectomy did not believe in their personal ability to withstand surgery and did not believe in the benefits of surgery. It could also be due to a lack of effective counseling. This is controversial to the results of a study carried out in Eastern Nigeria where 50.7% of patients accepted mastectomy and the main reason for refusal was the cost of the surgery [12]. 24.4% of the patients missed a dose of their chemotherapy without any medical indication. This could be due to cost or preference for other treatment options. It could also simply be that the symptoms got better.

From the data analysis, this result had a significant association with adherence (P=0.029). Of the 82 patients who took part in the study, 80.5% had an interest in alternative treatment: prayers (34%), traditional medicine (26%), and Chinese medicine (5%). This could be due to misconceptions about the recommended treatment though it had no significant relationship with adherence to recommended treatment. This is related to the results of a study carried out to better understand the specific reasons for acceptance or refusal of chemotherapy in older adults with cancer where it was discovered that Interest in complementary/alternative medicine showed no significant association with adherence to chemotherapy [17]. This result is different from the result of a retrospective observational study carried out to compare adherence to treatment and characteristics of patients receiving conventional cancer therapy with or without complementary medicine. This later study revealed that patients who received complementary medicine were more likely to refuse conventional cancer therapy and had a higher risk of death [18]. This difference could be due to the small sample size in the present study. For those who gave reason for non-adherence, the most prominent reason was medication cost (48.8%) followed by fear (29.3%). In contrast, another similar study revealed that forgetfulness (41.3%) and intolerance of side effects (36.5%) were the most common reasons for non-adherence [19]. This could be due to differences in socioeconomic status in different parts of the world.

Overall, 75.6% perceived their disease as severe. From the HBM, their perception of the severity of their disease is a strong push to cause them to accept and adhere to recommended treatment. The low rate of adherence despite the perceived severity could be due to the absence of other motivating factors. 61% of the patients believed in their susceptibility to developing serious complications if their cancer is not treated and 53.7% perceived their treatment as beneficial. The low rate of adherence despite a high perceived severity could also be explained by the low perceived treatment benefit of 53.7%. The study also revealed that 42.7% of the patients were unsatisfied with the information received from their health care provider. From the data analysis, there was no significant relationship between the patient's perception and adherence; perceived severity (P=0.266), perceived susceptibility (P=0.35), and perceived benefits (P=0.48).

The study revealed that 39.0% of patients had a low motivation score. The patient's motivation showed a significant relationship with adherence to chemotherapy. Patients with high motivation were more likely to adhere to treatment than patients with low motivation (P=0.0001). This is similar to the results in investigating the effect of motivation on adherence to Anti-Retroviral Treatment (ART) amongst HIV patients, which revealed that optimal ART adherence was significantly associated with higher motivation to adhere to ART (OR=1.171, P=0.011) [20]. This could be due to the absence of treatment reminder measures such as phone calls, treatment schedules, and home visits. More than forty percent 41.5% of the patients had a low knowledge score. The data analysis showed a significant relationship between patient´s knowledge and adherence to chemotherapy. Patients with high knowledge were more likely to adhere to treatment than patients with low knowledge (P=0.0001). This is similar to the results of an earlier study carried out to investigate the relationship between knowledge and adherence to pharmaceutical treatment in patients with hypertension. This earlier study revealed that the low knowledge level group included significantly more low-adherence patients than the group with a high level of knowledge (P=0.023) [21]. This could be due to a lack of proper information on treatment benefits and a lack of public awareness programs. In a medication adherence study in Saudi Arabia, it was revealed that low adherers viewed that the information they have received regarding their medications is less adequate when compared to patients with high adherence (P<0.001) [18,22].

## Conclusion

Although surgery and chemotherapy have been proven beneficial in the management of BC by preventing recurrence and extending survival, adherence is low and death rates due to this disease are on the increase especially in poor and developing countries. Results obtained showed adherence to surgery and chemotherapy amongst BC patients in MBH is low and is driven by the patient's motivation, knowledge towards the disease, and treatment. Community and hospital-based educational programs focused on addressing miss conceptions about treatment and treatment outcomes and strengthen counseling can help reduce nonadherence. More Extensive data are needed to confirm these findings.

### What is known about this topic

- Cancer medication non-adherence has been shown to lead to decreased survival, higher recurrence, treatment failure rates, and health care costs;
- Adherence to BC treatment is crucial to obtain optimal health outcomes, such as cure or improvement in the quality of life;
- Although most BC patients present late at diagnosis, survival rates can be greatly improved with improvement in adherence to recommended treatment.

### What this study adds

- Adherence to surgery and chemotherapy amongst BC patients in MBH is low and is driven by the patient's motivation, knowledge towards the disease, and treatment;
- Community and hospital-based educational programs focused on addressing miss conceptions about treatment and treatment outcomes and strengthen counseling can help reduce nonadherence.
